# Association between Protein Intake and the Risk of Hypertension among Chinese Men and Women: A Longitudinal Study

**DOI:** 10.3390/nu14061276

**Published:** 2022-03-17

**Authors:** Jingjing He, Siwang Yu, Aiping Fang, Xin Shen, Keji Li

**Affiliations:** 1 School of Pharmaceutical Sciences, Peking University Health Science Center, Beijing 100191, China; hejingjing@bjmu.edu.cn (J.H.); swang_yu@hsc.pku.edu.cn (S.Y.); 2 School of Public Health, Sun Yat-Sen University, Guangzhou 510275, China; fangaiping2006@126.com; 3 Peking University Cancer Hospital, Beijing 100142, China; shenxin19870918@163.com; 4 School of Public Health, Peking University Health Science Centre, Beijing 100191, China

**Keywords:** hypertension, plant protein, animal protein, total protein, China Health and Nutrition Survey

## Abstract

This study aimed to examine the relationship between hypertension risk and protein intake in Chinese individuals. Our analysis included 7007 men and 7752 women from 9 China Health and Nutrition Survey waves (1991–2015). The main outcome was incident hypertension. Dietary intake was recorded using a combination of 3 consecutive 24-h recalls and a household food inventory survey. Energy-adjusted cumulative average intakes were analyzed, and Cox proportional hazards regression models were built. After 143,035 person-years of follow-up, 2586 and 2376 new male and female hypertension cases were identified, respectively. In multivariate-adjusted models with dietary protein intakes included as categorical variables, higher animal protein intake was associated with lower hypertension risk in women (*p*-trend = 0.01), whereas non-significant in men. Plant protein intake showed a significant positive correlation with hypertension risk, while non-significant for total protein. On a continuous scale, restricted cubic spline curves visually revealed L-, J-, and U-shaped associations between hypertension risk and animal-, plant-, and total-protein intakes, respectively, in both sexes (all *p*-nonlinearity < 0.0001). Our results suggest a beneficial association between intakes of animal, plant, and total proteins and hypertension risk at lower intake levels, and excessive intake of plant or total protein may increase the hypertension risk in the Chinese population.

## 1. Introduction

Diet plays a prominent role in blood pressure (BP) homeostasis and the development of hypertension (HT). Dietary alterations have been recommended as important lifestyle changes to control BP and prevent HT [1]. In addition to well-established dietary factors that lower BP (e.g., weight, salt, and alcohol reduction/limitation) [2], interest in the association between dietary protein intake and BP or HT has grown in recent years [3].

Dietary protein consumption, particularly animal sources, has long been assumed to increase BP through adverse renal effects [4]. Since the 1980s, substantial research suggested that dietary protein might benefit BP, but results remain inconclusive [5]. Two recent systematic reviews found that most cross-sectional studies showed a weak inverse correlation between total protein intake and BP, with the pooled results being significant; however, the results of prospective cohort studies were inconclusive, and the pooled effect was not significant [6,7].

Besides the total protein intake, another concern is the relationship between BP or HT and protein from different sources (animal vs. plant). The traditional view is that plant protein intake decreases, whereas animal protein intake increases, BP [8]. Nevertheless, observational studies have indicated that the association between animal protein intake and BP or incident HT might be positive [9,10], negative [11], or absent [12,13]. A meta-analysis, pooling data from cross-sectional studies, showed a non-significant negative relation between plant, but not animal protein, and BP, while data from prospective studies showed that neither protein source significantly correlated with incident HT [7]. Results from intervention studies reveal no differential BP effects from either protein source [14].

In brief, cross-sectional studies have shown a relatively consistent negative correlation between total protein, plant protein, and BP, but prospective studies have not confirmed this result. The observed effects of animal protein intake on BP or incident HT are mixed, and no definite conclusions can be drawn. Additionally, relevant large prospective studies were mostly conducted in Western populations. Regarding Eastern populations, only one Japanese study has been performed, but it did not distinguish between the effects of animal and plant proteins [15].

Eastern and Western populations consume animal- and plant-based diets, respectively, resulting in differences in the protein composition of the diets. Researchers have speculated that the benefits of plant versus animal protein on BP or HT might be more pronounced in populations consuming more Westernized diets [7]. The limited available results necessitate evidence from longitudinal epidemiological studies in Eastern populations, such as China, to verify these findings in Western populations. Accordingly, this study aimed to explore the association between dietary protein from different sources and the risk of incident HT in a cohort of Chinese adults.

## 2. Materials and Methods

### 2.1. Study Population

Data from the present study were obtained from the China Health and Nutrition Survey (CHNS), an ongoing, open, prospective cohort study in China. The CHNS was initiated in 1989 and has completed a total of 9 rounds of follow-up in 1991, 1993, 1997, 2000, 2004, 2006, 2009, 2011, and 2015. The survey was approved by the institutional review boards at the University of North Carolina, Chapel Hill, NC, USA, and the National Institute of Nutrition and Food Safety, China Centre for Disease Control and Prevention. All participants provided written informed consent for participation. Details of the CHNS have been described elsewhere [16,17].

**Figure 1:**
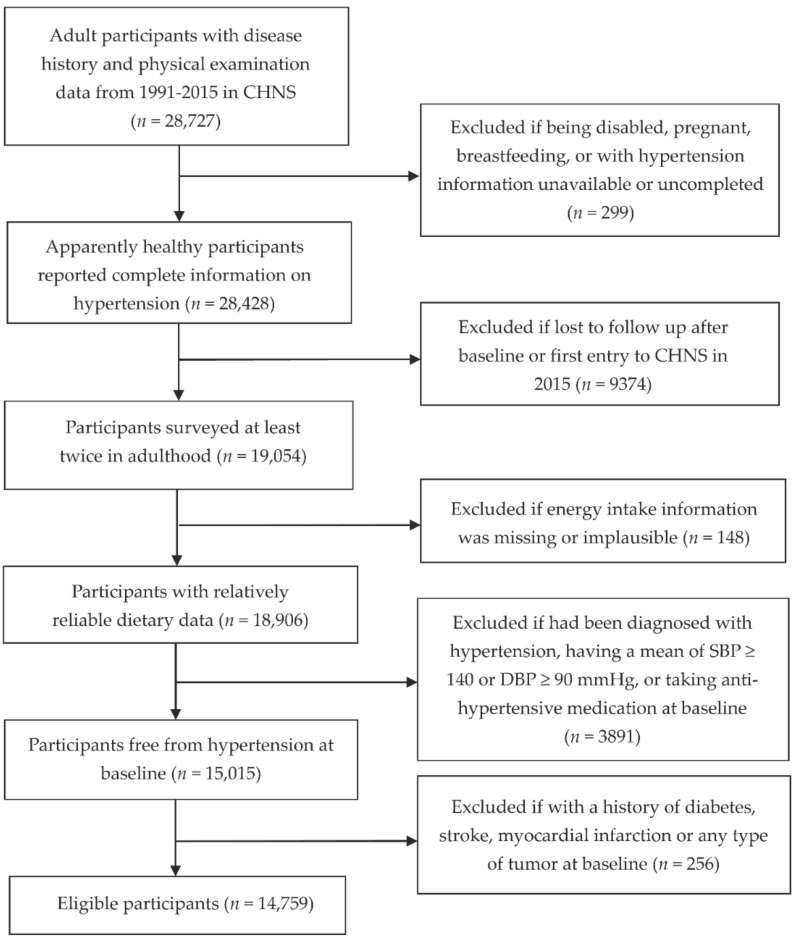
Flow chart of participant inclusion.

We used data from 28,727 adult participants aged 18 years or older with a disease history and with physical examination data available from 1991 to 2015 in the CHNS. We excluded participants who were pregnant, nursing, or disabled, had unavailable or incomplete HT information, or were lost to follow-up after the baseline survey or first entered the survey in 2015. We also excluded participants with missing or implausible total energy (TE) intake information (>5000 kcal/day or <700 kcal/day); those who had been diagnosed with HT, having a mean systolic BP (SBP) of ≥140 mmHg or diastolic BP (DBP) of ≥90 mmHg or who were taking antihypertensive medication at baseline; and those with a history of diabetes, stroke, myocardial infarction, or any type of tumor at baseline. Finally, 7007 men and 7752 women were included in the analysis (with ages, SBP, and DBP ranging from 18 to 84 years, 78.0 to 139.3 mmHg, and 50.0 to 89.3 mmHg, respectively) (Figure 1).

### 2.2. Ascertainment of Outcome

Experienced health workers measured BP on the right arm of participants after they had sat down and rested for at least 5 min. Standard calibrated mercury sphygmomanometers were used, and standardized procedures were followed. The Korotkoff method was used to determine SBP (Korotkoff phase 1) and DBP (Korotkoff phase 5). For each subject, BP was measured three times, 30 s apart. We calculated the average of the three readings for analysis [18,19].

Since 1997, a questionnaire-based interview has also been conducted as part of each survey to ask the participants about their history of HT. The questions were posed in the following manner: (1) “Has a doctor ever told you that you have high BP?” If yes, (2) “For how many years have you had high BP?” and (3) “Are you currently taking anti-HT drugs?” Information on the diagnosis of HT prior to 1997 was indirectly deduced from the answers obtained in subsequent questionnaires that were returned by the same individual. If multiple or inconsistent records were present, we retained only the first record to minimize recall bias.

Accordingly, incident HT, our main outcome variable, was ascertained by meeting at least one of three criteria: (1) SBP ≥ 140 mmHg or DBP ≥ 90 mmHg; (2) a new physician diagnosis of HT; and (3) taking anti-HT medication.

### 2.3. Measurement of Dietary Intakes

Details of the dietary intake methods used in the CHNS have been published previously [20]. The methods comprised a combination of 3 consecutive 24-h dietary recalls and a household food inventory survey. At the individual level, each participant was asked to report all food consumed at home and away from home for 3 consecutive days using a 24-h recall. At the household level, all foods and condiments stored, purchased, picked, and wasted were carefully weighed and recorded over the same 3 consecutive days; the changes in household storage were calculated and considered as household food consumption.

The household food inventory survey was used as an addition to the individual 24-h recall method. Based on the household consumption and the proportion of individual TE intake to the TE intake of all household members, we calculated the individual daily consumption of cooking oils and condiments [19]. We subsequently added it to the 24-h dietary recall data to obtain a more accurate measure of individual dietary intake. The combined dietary intake method used in this survey has been validated for TE intake using the doubly labeled water method [21].

The intake of TE and nutrients was estimated using the Chinese Food Composition Tables. Three-day average intakes were used in the analysis. Total protein, animal protein, and plant protein were the main exposure variables considered. Animal protein was defined as protein from meat, fish, eggs, and dairy products. Plant protein included protein from cereals, tubers, vegetables, fruits, soya, legumes, and nuts. After log transformation, we adjusted the intake of each nutrient according to the TE intake of men (2451 kcal/day) and women (2090 kcal/day) (mean TE intake of adults in the survey) using the residual method [22]. Additionally, to reduce within-subject variation and represent long-term habitual dietary intakes, we calculated the cumulative average intake of each nutrient obtained from all dietary measures in each survey round before the outcome was identified [23].

### 2.4. Assessment of Non-Dietary Covariates

Well-trained interviewers followed standardized measuring and survey procedures to collect information on non-dietary covariates, including anthropometric, sociodemographic, and lifestyle variables. Body weight and height were measured using certified scales and stadiometers, with the readings recorded to the nearest 0.1 kg and 0.1 cm, respectively. Body mass index (BMI) was calculated as weight in kilograms (kg) divided by the square of the height in meters (m^2^). Age was recorded as an integer in years. Residential area was classified as rural or urban. The highest education level was divided into three categories: low (primary school and lower), medium (lower or upper-middle school and technical or vocational school), and high (college, university, and higher). Per capita annual household income was quartered as low, medium, high, and very high. The self-reported physical activity level (PAL, mainly occupational) was quantized into multiples of basal metabolic rate (BMR): 1.3 × BMR for “very light” (for both sexes), and 1.6 and 1.5 × BMR for “light”, 1.7 and 1.6 × BMR for “moderate”, 2.1 and 1.9 × BMR for “heavy”, and 2.4 and 2.2 × BMR for “very heavy” in men and women, respectively. Furthermore, smoking status (former or current, or never) and alcohol consumption (yes or no) were treated as binary variables. For all non-dietary covariates, the measurement in the baseline year was used for analysis.

### 2.5. Statistical Analysis

All analyses were performed separately for men and women. We presented the characteristics of participants according to the age-specific quintiles of dietary protein intake. We calculated means (±standard deviations) and medians (interquartile ranges) for continuous variables and counts (percentages) for categorical variables. Linear regression (with the median intakes of each quintile included in the model) was used to test linear trends for continuous variables, and the chi-square with linear-by-linear association test was used for categorical variables.

For each subject, the baseline referred to the year of the subject’s first entry into the survey with a complete dietary record. Participants contributed to follow-up person-time from baseline up to the first HT diagnosis, the last survey round before the subject’s departure from the CHNS, or the latest survey (2015), whichever came first. The number of new HT cases was divided by person-years of follow-up in each quintile to calculate the incidence rates.

We built Cox proportional hazards regression models to estimate hazard ratios (HRs) and the corresponding 95% confidence intervals (CIs) for developing HT. Dietary protein intakes were categorized according to quintiles, and the lowest intake quintiles were used as references. The proportional hazards assumption was not violated by likelihood ratio tests assessing the significance of interaction terms of categories of intakes and log-transformed follow-up time.

Cox regression models were stratified according to the year participants entered the study and were adjusted for potential confounders, which were selected from previous prospective studies exploring the protein–HT association. Considering that the collinearity of covariates may confound results, we conducted a correlation analysis among nutritional variables before our formal analysis. We eliminated variables that were highly correlated with our main exposure variables in the final models (e.g., cholesterol and CHO intakes, found to be highly related to animal protein (r = 0.69) and plant protein (r = 0.68) intakes, respectively). Additionally, given that dietary saturated fat (SFA) and monounsaturated fat (MUFA) (r = 0.84), as well as magnesium and potassium (r = 0.62), were highly related in our data, we eventually adjusted for SFA and magnesium, but not for MUFA and potassium, in our analysis because it produced better goodness of fit of the models.

Adjustment for confounders was performed sequentially using the three models. Model 1 was adjusted for age, BMI, and dietary intake of TE. Model 2 was further adjusted for other non-dietary factors, including residence area, highest education level, household income level, PAL, smoking status, alcohol consumption, and SBP at baseline. Model 3 was further adjusted for dietary intakes of SFA, polyunsaturated fat (PUFA), dietary fiber, sodium, calcium, and magnesium. In addition, mutual adjustment was performed for dietary animal protein and plant protein intakes in Model 3. Tests for trends of HRs were conducted using the median value for each quintile of intake as a continuous variable.

To evaluate the potential effect modification, analyses were further stratified by some known HT risk factors, including age (<50 or ≥50 years), BMI (<24 or ≥24 kg/m^2^), smoking status (former or current, or never), and alcohol consumption (yes or no). Each dichotomized variable was multiplied by the median value of protein intake (g/day) to generate a multiplicative term, which was used to assess the interaction with a likelihood ratio test.

Moreover, the dose-response associations between dietary protein intakes and HT risk were evaluated on a continuous scale with restricted cubic spline (RCS) curves based on the multivariate-adjusted Cox proportional hazards models Model 3s. The 10th, 50th, and 90th percentiles were retained as knots, and the median intakes were set as the references. The SAS macro program %RCS_Reg for curve fitting was provided by Loic Desquilbet and François Mariotti [24].

All *p*-values were two-sided, and statistical significance was set at *p* < 0.05. All analyses were conducted using SAS (version 9.4; SAS Institute Inc., Cary, NC, USA) and SPSS for Windows (version 20.0; IBM Corp., Armonk, NY, USA).

## 3. Results

The present analysis included 14,759 subjects. During 143,035 person-years of follow-up (median [IQR]: 7 [4,15] years), we identified 2586 and 2376 new cases of HT in 7007 men and 7752 women, respectively. At baseline, the median age was 35 years for both sexes, while it was 50 and 54 years for men and women at the first HT diagnosis, respectively. The median intakes of total, animal, and plant protein were 73.3 (12.0), 18.9 (3.1), and 50.5 (8.2) g/day (%TE) among men, and 63.4 (12.2), 16.5 (3.2), and 43.7 (8.4) g/day (%TE) among women, respectively. On average, animal protein accounted for 26.7% of the total protein intake in all participants in our analysis.

Table 1 and Table 2 present the characteristics of dietary intake and baseline non-dietary factors in men and women, respectively. At baseline, men and women with a higher animal protein intake had a higher BMI, higher SBP, lower PAL, higher education and income levels, were more likely to be urban residents and alcohol consumers, and less likely to be smokers. For plant protein intake, extensive opposite associations (versus animal protein) were observed for non-dietary factors, with a few exceptions that showed non-significant associations (e.g., smoking and drinking status among men). In both sexes, the intakes of most other nutrients increased with the quintiles of animal protein intake, except that of TE, plant protein, CHO, dietary fiber, sodium, and magnesium, which decreased. With increased quintiles of plant protein intake, the intakes of most other nutrients also increased, except for the intakes of animal protein, SFA, PUFA, MUFA, and cholesterol, which decreased. In addition, sodium consumption was not significantly different across quintiles of plant protein intake.

Table 3 shows the association between HT risk and protein intake with protein intake included as priori defined quintile categorical variables in the models. After adjusting for non-dietary and dietary factors, we observed that animal protein intake was inversely associated with the risk of HT in women (HR [95% CI] of the highest quintile versus the lowest was 0.76 [0.62–0.93], *p*-trend = 0.010). Among men, the HRs (1.00, 0.75, 0.73, 0.77, 0.78) across quintiles of animal protein intake revealed a negative association, but the *p*-trend value (0.137) showed a non-significant linear trend. We further included red meat (i.e., livestock meat) protein and white meat (i.e., poultry and fish meat) protein into separate models to analyze their respective associations with HT (Table S1). In women, red meat protein intake was significantly inversely associated with HT risk (*p*-trend = 0.044), whereas white meat protein showed a marginally significant inverse association (*p*-trend = 0.059). In men, only white meat protein showed a significant negative association with HT (*p*-trend = 0.018).

A significant positive correlation was found between plant protein and HT risk, with multivariate adjusted HRs (95% CIs) across the first to fifth quintiles of 1.00, 0.83 (0.72–0.95), 0.78 (0.67–0.91), 0.93 (0.79–1.10), and 1.11 (0.91–1.36) (*p*-trend = 0.031) in men, and 1.00, 0.89 (0.76–1.05), 0.92 (0.77–1.08), 1.07 (0.89–1.28), and 1.29 (1.04–1.59) (*p*-trend = 0.0003) in women, respectively. Analysis of total protein intake suggested a non-significant linear association with HT risk, but the HRs from the lower quintiles predicted a possible negative association in both sexes.

Although the above analysis of including intakes as categorical variables into models yielded many significant results, by looking at the changes of HR across intake quintiles, we found that most of the changes were fluctuating rather than linear, suggesting some possible non-linear relationship. Therefore, we performed further analysis using RCS models by including intakes as continuous variables.

Table S2 presents the results of the effect modification analysis. No significant interaction was observed in our stratified analysis, except that among women, the negative animal protein–HT association was pronounced among participants with a normal weight rather than among those who were overweight or obese (*p*-interaction = 0.003). The HR for the highest versus lowest quintile of animal protein intake was 0.70 (95% CI: 0.55–0.91) and 0.94 (95% CI: 0.66–1.33) in women with a normal weight and those who were overweight or obese, respectively.

**Figure 2:**
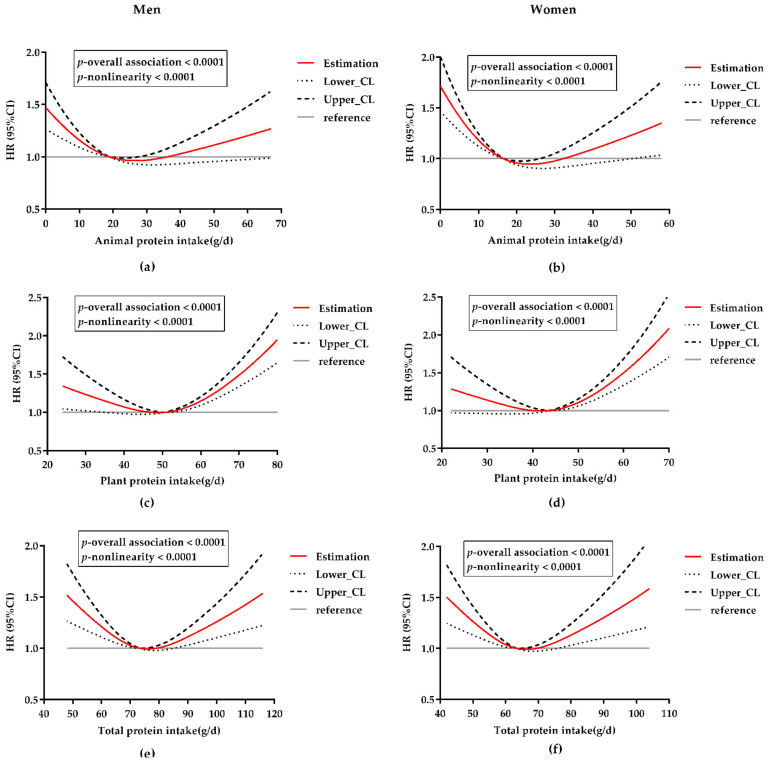
Multivariable-adjusted HRs and 95% CIs for the risk of HT according to dietary intakes of animal, plant, and total protein on a continuous scale. CI, confidence interval; CL, confidence limit; HT, hypertension. (**a**) Association between animal protein intake and HT risk in men; (**b**) Association between animal protein intake and HT risk in women; (**c**) Association between plant protein intake and HT risk in men; (**d**) Association between plant protein intake and HT risk in women; (**e**) Association between total protein intake and HT risk in men; (**f**) Association between total protein intake and HT risk in women. Red solid lines represent the multivariable-adjusted HRs, with black dotted lines representing the 95% CIs. Gray horizontal solid lines represent the references that were set at the median intakes, corresponding to an HR of 1.0. Analyses were adjusted for a series of variables according to Model 3.

In Figure 2, we used RCSs to construct a flexible model and visualize the relationship between the risk of HT and protein intake on a continuous scale. Significant non-linear associations between animal, plant, total protein intake, and HT risk were observed in both men and women. All *p*-values for nonlinearity were less than 0.0001.

For total protein (Figure 2e,f), the curves were typically U-shaped in both sexes. The U-shaped curves indicate that total protein intake was inversely associated with HT risk at lower intake levels, but higher intakes increased the risk when exceeding a certain threshold. That is to say, a total protein intake that is too low or too high might increase the HT risk.

For animal protein and plant protein, the curves were similar to that of total protein, although each had its own characteristics. The 95% CI curves indicated that the latter half of the curves for animal protein were not significant relative to the median intake, which led to an approximately L-shaped animal protein–HT association (Figure 2a,b). We further analyzed the respective associations of red meat protein and white meat protein with HT by including the respective intakes as continuous variables in models. The results for both red and white meat proteins were consistent with the results for animal protein (Figure S1). The L-shaped curves mean that animal protein (including red meat and white meat protein) intake was inversely associated with HT risk at lower intake levels, while at higher intake levels, there was no significant association in our analysis.

Correspondingly, the relationship between vegetable protein intake and HT was mainly J-shaped (Figure 2c,d), given that the first half of the curve was relatively flat, and it was not significant among women according to the dotted lines of the CIs. The J-shaped curves indicate an inverse plant protein–HT association (significant in men, not women) when intakes were at lower levels, while the excessive intake was significantly positively associated with HT risk at higher levels.

In men and women, the lowest risks were observed for intakes of 26.5 g/day and 23.5 g/day for animal protein, 48.8 g/day and 42.1 g/day for plant protein, and 76.2 g/day and 66.2 g/day for total protein, respectively, which were all close to the population’s median intakes.

## 4. Discussion

### 4.1. Summary

Our study prospectively explored the association between dietary protein from different sources and HT risk among a Chinese population whose dietary protein was mainly plant-derived (73.3% of the total protein intake). We found that the risk patterns of HT with dietary protein consumption were L-, J-, and U-shaped for the intakes of animal, plant, and total protein, respectively.

### 4.2. Comparison with Other Studies

To the best of our knowledge, prospective studies in this field were mostly among Western populations. Regarding the association between animal-protein intake and HT risk or BP, these studies have yielded conflicting results, which reported positive [9,10,25], negative [11,26], or non-significant [13,27,28] associations, respectively. In our study, animal protein was negatively associated with HT risk at a relatively low level of intake. When the intake exceeded a certain threshold, the HT risk increased non-significantly. No prospective studies have explored protein–HT association in the Chinese population; nonetheless, some cross-sectional studies [29,30,31] have reported negative correlations between animal protein and BP. It has been assumed that an inverse animal protein–BP relationship may be found more frequently in populations with a relatively low animal protein intake [13,30]; this interpretation also applies to our findings.

Prospective studies had different findings regarding the plant protein–HT association. Studies conducted in Western populations [9,11,13] reported that plant protein intake was inversely related to changes in BP or HT risk, while others (also among Western populations) [10,25,26,27] failed to detect significant associations. Similarly, we found a possible protective effect of plant protein against HT risk within the lower-intake range, although this was not pronounced in women.

Surprisingly, we observed a significant positive plant protein–HT association after the intake thresholds; this has not been observed in previous prospective studies. The significantly higher plant protein intake in our population may explain this new finding. Among Western populations, plant protein intakes were approximately 3.5–5.2%TE [9,13,28] (26.2 g/day in the Netherlands [28]) on average, and the mean of the highest intake quartile was 29.6 g/day in the US [26]. In our study, men and women consumed 50.5 g/day (8.2%TE) and 43.7 g/day (8.4%TE) of plant protein, respectively, where the lowest intake quintiles (35.3 g/day in men and 30.9 g/day in women) were compatible with the highest intake quartile in the US. Western plant protein intakes are probably insufficiently high to increase HT risk. Furthermore, corroborating our findings, a cross-sectional study in the Japanese population [32], with a plant protein intake (Q1–Q4: 24.9, 33.3, 40.5, and 54.9 g/day) close to that of the Chinese population and much higher than that of Western populations, reported a positive association between plant protein intake and SBP.

Regarding the total protein–HT correlation, most previous prospective studies have reported non-significant results, while two studies in the US [11,26] and one in Japan [15] have found negative associations; another US study [9] found positive associations. In the present analysis, the role of total protein was the result of the combined effects of animal and plant proteins. The negative correlation revealed by the first half of the curve was consistent with our findings for animal and plant proteins. The positive correlation in the second half should be attributed to plant protein, which contributed the majority of total protein intake.

### 4.3. Possible Explanations and Implications

Our study provides evidence that both animal and plant proteins have a favorable effect on HT among the lower-intake ranges. A possible assumption is that some amino acids produced via protein digestion benefit BP control. As an amino acid rich in both animal and plant foods, arginine may dilate the blood vessels by producing nitric oxide and act as an antioxidant by regulating redox-sensitive proteins, thereby having an antihypertensive effect [33,34]. Tryptophan and tyrosine, acting as precursors for the synthesis of 5-hydroxytryptamine (5-HT) and norepinephrine, are also found in many plant and animal sources, which contribute to lower BP through 5-HT formation pathways, central catecholamine action on alpha-receptors, or interference with the renin–angiotensin axis [1,35,36].

At higher levels, increased plant- and total-protein intakes tended to increase HT risk, based on our analysis. It was proposed that infiltrating immune cells induced by a high protein load may exert deleterious effects by releasing free radicals, cytokines, or other vasoactive factors in the kidney, blood vessels, brain, or other organs, thereby increasing BP [37]. Calcium plays an important role in BP regulation [38]. Increased protein intake has been found to induce the kidneys to excrete calcium [39], thus indirectly increasing BP.

We showed excessively low and high protein intakes to be related to an increased HT risk, which differs from reported positive, negative, or null associations. Therefore, the generally accepted high-protein diet for weight loss should be carefully considered, supported by findings that long-term consumption of high-protein diets, irrespective of the protein source, in those with normal renal function may cause renal injury [40]. Furthermore, compared to results from Western prospective studies, we found interesting commonalities and differences. Nutrients that were consumed more often in one population (i.e., animal and plant protein in Western and Chinese diets, respectively) tended to cause risks at high intake levels, while other nutrients with a lower habitual intake (i.e., plant and animal protein in Western and Chinese diets, respectively) were relatively safe. Hence, maintaining a balanced diet (i.e., containing adequate amounts and proportions of animal and plant protein) may be more beneficial to health and is essential for reducing chronic disease risk [31]. Therefore, population-specific dietary or nutritional characteristics should be considered for future planning of targeted and practical dietary suggestions.

### 4.4. Strengths and Weaknesses of Our Study

This study had several strengths. No previous prospective study has investigated the protein–HT association in the Chinese population, and this is one of the very few long-term prospective studies exploring the respective effects of animal and plant proteins on HT risk in Asian populations. Repeated dietary assessments enabled us to obtain a relatively accurate estimate of long-term habitual dietary intake. Furthermore, our analysis controlled for various dietary and non-dietary covariates to reduce confounding effects. Additionally, we used RCS to illustrate the dose-response relationship further and found non-linear associations. Nevertheless, there were also certain limitations. First, information on some important factors that may potentially be associated with HT risk, such as family HT history and corticosteroid use, were not available in our study; due to these limitations, we could not reach a sound conclusion. Second, despite the large sample size, this participant sample was not representative of the general Chinese population, limiting our findings’ external validity. Third, although we adjusted for several relevant risk factors, we cannot rule out the possibility of residual confounding due to the nature of the observational study.

### 4.5. Future Research

Prospective studies should be carried out in different populations consuming plant-based diets to provide comparisons and to verify our findings. Moreover, studies investigating the mechanisms elucidating the potential beneficial or harmful effects of protein intake on BP or HT are needed.

## 5. Conclusions

We found non-linear associations between the dietary intake of animal, plant, and total protein and HT risk. This confirmed the previously reported beneficial effect of animal and plant proteins on HT, although only at lower intake levels. Our findings also suggest that excessive intake of either plant or total protein may increase the risk of HT in the Chinese population. Overall, our findings underscore the need for maintaining a balanced diet containing adequate amounts and proportions of animal and plant protein, which may be more beneficial to health and chronic disease risk reduction.

## Figures and Tables

**Table 1:** Baseline sociodemographic and lifestyle factors and cumulative average dietary intakes in 7007 men according to quintiles of animal and plant protein intake ^1^.

	Quintiles of Animal Protein Intake					*p* for Trend	Quintiles of Plant Protein Intake					*p* for Trend
	1	2	3	4	5		1	2	3	4	5	
No. of participants	1401	1401	1402	1401	1402		1401	1401	1402	1401	1402	
Age (year)	37.4 ± 15.4	36.4 ± 14	37.3 ± 14.3	37.2 ± 14.3	37.2 ± 14.1	0.7711	38.6 ± 14.8	36.5 ± 14	37.3 ± 14.1	36.0 ± 14.2	37.0 ± 14.9	0.003
BMI (kg/m^2^)	21.3 ± 2.6	21.6 ± 2.7	21.8 ± 2.8	22 ± 2.9	22.5 ± 3.4	<0.0001	22.3 ± 3.3	21.8 ± 3.0	21.7 ± 3.0	21.6 ± 2.7	21.7 ± 2.6	<0.0001
PAL (×BMR)	2.0 ± 0.2	1.9 ± 0.3	1.8 ± 0.3	1.7 ± 0.3	1.6 ± 0.3	<0.0001	1.6 ± 0.3	1.7 ± 0.3	1.8 ± 0.3	1.9 ± 0.3	1.9 ± 0.3	<0.0001
Urban residential area, *n* (%)	162 (11.6%)	335 (23.9%)	487 (34.7%)	657 (46.9%)	891 (63.6%)	<0.0001	766 (54.7%)	593 (42.3%)	496 (35.4%)	405 (28.9%)	272 (19.4%)	<0.0001
Education level, *n* (%)						<0.0001						<0.0001
Low	753 (53.7%)	608 (43.4%)	449 (32%)	332 (23.7%)	214 (15.3%)		308 (22.0%)	392 (28.0%)	489 (34.9%)	524 (37.4%)	643 (45.9%)	
Medium	640 (45.7%)	758 (54.1%)	871 (62.1%)	945 (67.5%)	939 (67%)		872 (62.2%)	898 (64.1%)	831 (59.3%)	823 (58.7%)	729 (52.0%)	
High	8 (0.6%)	35 (2.5%)	82 (5.8%)	124 (8.9%)	249 (17.8%)		221 (15.8%)	111 (7.9%)	82 (5.8%)	54 (3.9%)	30 (2.1%)	
Household income level, *n* (%)						<0.0001						<0.0001
Low	807 (57.6%)	560 (40.0%)	378 (27.0%)	247 (17.6%)	119 (8.5%)		166 (11.8%)	305 (21.8%)	429 (30.6%)	519 (37.0%)	692 (49.4%)	
Medium	377 (26.9%)	470 (33.5%)	435 (31.0%)	402 (28.7%)	296 (21.1%)		265 (18.9%)	426 (30.4%)	437 (31.2%)	430 (30.7%)	422 (30.1%)	
High	165 (11.8%)	234 (16.7%)	364 (26.0%)	402 (28.7%)	407 (29.0%)		399 (28.5%)	376 (26.8%)	321 (22.9%)	287 (20.5%)	189 (13.5%)	
Very high	52 (3.7%)	137 (9.8%)	225 (16.0%)	350 (25.0%)	580 (41.4%)		571 (40.8%)	294 (21.0%)	215 (15.3%)	165 (11.8%)	99 (7.1%)	
Ever and current smoker, *n* (%)	931 (66.5%)	903 (64.5%)	868 (61.9%)	841 (60.0%)	845 (60.3%)	<0.0001	894 (63.8%)	859 (61.3%)	866 (61.8%)	865 (61.7%)	904 (64.5%)	0.666
Alcohol consumer, *n* (%)	840 (60.0%)	835 (59.6%)	900 (64.2%)	858 (61.2%)	888 (63.3%)	0.041	895 (63.9%)	848 (60.5%)	867 (61.8%)	867 (61.9%)	844 (60.2%)	0.143
SBP (mm Hg)	113.0 ± 11.5	113.0 ± 11.3	114.1 ± 10.9	114.2 ± 10.9	115.8 ± 10.5	<0.0001	115.6 ± 10.6	114.0 ± 11.2	113.3 ± 11.1	113.2 ± 10.8	113.9 ± 11.4	<0.0001
TE (kcal/day)	2665.0 ± 645.9	2512.1 ± 545.9	2485.4 ± 561	2465.6 ± 546.2	2404.2 ± 603.2	<0.0001	2436.0 ± 656.2	2471.2 ± 530.8	2483.1 ± 524.9	2497.8 ± 548.5	2644.0 ± 644.6	<0.0001
Protein (g/day)	67.8 ± 10.8	68.4 ± 9.5	71.2 ± 9.1	76.2 ± 8.5	89.5 ± 13.8	<0.0001	74.1 ± 16.2	73.5 ± 12.6	73.0 ± 12.3	73.4 ± 11.6	79.1 ± 11.9	<0.0001
Protein (%TE)	11.0 ± 1.7	11.2 ± 1.5	11.7 ± 1.5	12.5 ± 1.5	14.7 ± 2.4	<0.0001	12.2 ± 2.8	12.1 ± 2.2	12.0 ± 2.1	12.0 ± 2.0	12.9 ± 2.0	<0.0001
Animal protein (g/day)	1.9 (0.0, 4.1)	10.7 (8.6, 12.7)	18.9 (16.8, 20.9)	27.4 (25.2, 29.9)	41 (36.2, 48.2)	<0.0001	33.0 (24.1, 43.4)	25.5 (18.2, 32.9)	19.2 (11.5, 27.1)	12.2 (5.6, 20.6)	4.7 (0.5, 11.3)	<0.0001
Animal protein (%TE)	0.3 (0.0, 0.7)	1.7 (1.4, 2.1)	3.1 (2.7, 3.5)	4.5 (4.1, 5.0)	6.9 (6.0, 8.3)	<0.0001	5.5 (3.9, 7.3)	4.1 (3.0, 5.5)	3.1 (1.9, 4.5)	2.0 (0.9, 3.4)	0.8 (0.1, 1.8)	<0.0001
Plant protein (g/day)	63.6 ± 10.7	55.7 ± 9.7	50.0 ± 9.1	46.0 ± 8.5	41.8 ± 10.1	<0.0001	35.3 ± 5.7	44.7 ± 1.7	50.5 ± 1.7	57.2 ± 2.2	69.4 ± 7.3	<0.0001
Plant protein (%TE)	10.4 ± 1.8	9.1 ± 1.6	8.2 ± 1.5	7.5 ± 1.4	6.8 ± 1.6	<0.0001	5.8 ± 0.9	7.3 ± 0.3	8.2 ± 0.3	9.3 ± 0.4	11.3 ± 1.2	<0.0001
CHO (%TE)	71.3 ± 8.1	63.5 ± 7.9	58.2 ± 8.0	54.5 ± 7.8	49.2 ± 9.3	<0.0001	47.1 ± 9.3	56.1 ± 7.3	60.1 ± 7.9	64.3 ± 8.4	69.1 ± 8.6	<0.0001
SFA (%TE)	3.5 ± 2.3	5.6 ± 2.5	6.7 ± 2.4	7.7 ± 2.5	8.6 ± 2.8	<0.0001	9.3 ± 3.1	7.4 ± 2.3	6.4 ± 2.3	5.2 ± 2.2	3.7 ± 2.0	<0.0001
PUFA (%TE)	3.8 (2.4, 6.1)	4.6 (3.1, 7.3)	5.5 (3.6, 8.0)	5.8 (4.1, 8.2)	6.5 (4.6, 9.3)	<0.0001	6.9 (4.6, 10.1)	5.6 (3.8, 8.0)	5.0 (3.3, 8.0)	5.0 (3.0, 7.4)	4.1 (2.8, 6.2)	<0.0001
MUFA (%TE)	6.3 ± 3.5	9.6 ± 3.6	11.9 ± 3.7	13.5 ± 3.7	14.9 ± 4.5	<0.0001	16.2 ± 4.4	13.0 ± 3.4	11.1 ± 3.4	9.1 ± 3.4	6.7 ± 3.4	<0.0001
Dietary fiber (g/day)	14.7 (10.9, 18.1)	12.1 (9.4, 15.2)	10.5 (8.4, 13.5)	9.9 (7.9, 12.7)	9.8 (7.5, 13.2)	<0.0001	9.0 (7.0, 11.8)	9.6 (7.8, 12.3)	10.4 (8.5, 13)	12.0 (9.9, 14.8)	16.1 (13.4, 19.0)	<0.0001
Cholesterol (mg/day)	25.6 (0.0, 65.4)	126.8 (79.3, 194.0)	195.8 (130.4, 286.7)	258.2 (190.0, 355.7)	356.2 (269.5, 489.2)	<0.0001	295.9 (201.5, 422.7)	239.7 (154.2, 343.0)	193.7 (116.7, 292.4)	135.9 (64.5, 236.3)	60.0 (11.3, 156.4)	<0.0001
Sodium (mg/day)	6369 ± 3024	6473 ± 2835	6094 ± 2843	6095 ± 2631	6060 ± 2914	<0.0001	6332 ± 3247	5955 ± 2470	6219 ± 2706	6256 ± 2772	6327 ± 3012	0.240
Calcium (mg/day)	405.4 ± 173.1	411.8 ± 164.4	414.0 ± 155.0	424.4 ± 162.9	494.7 ± 266.4	<0.0001	409.1 ± 189.9	413.5 ± 154.8	422.7 ± 220.8	434.8 ± 167.7	470.4 ± 211.0	<0.0001
Magnesium (mg/day)	389.2 ± 100.4	345.6 ± 78.1	325.6 ± 68.0	320.6 ± 63.6	326.2 ± 69.1	<0.0001	286.5 ± 57.5	312.4 ± 52.8	329.5 ± 58.6	362.5 ± 69.6	416.3 ± 91.7	<0.0001
Potassium (mg/day)	1786.9 ± 547.4	1670.1 ± 450.5	1671.8 ± 410.6	1734.0 ± 381.4	1935.2 ± 483.9	<0.0001	1700.7 ± 446.2	1689.5 ± 401.3	1698.0 ± 383.5	1773.4 ± 409.6	1936.2 ± 616.7	<0.0001

^1^ Values are presented as the mean ± SD, count (%), or median (IQR). We calculated dietary intake as the energy-adjusted cumulative average of baseline and follow-up data in men and women, respectively. For non-dietary factors, we used the baseline measurement. To test the linear trends, we used linear regression for continuous variables (including the median intake of animal or plant protein as continuous variables in the regression models) and the chi-square with linear-by-linear association tests for categorical variables. BMI, body mass index; BMR, basal metabolic rate; CHO, carbohydrates; IQR, interquartile range; MUFA, monounsaturated fat; PAL, physical activity level; PUFA, polyunsaturated fat; SD, standard deviation; SBP, systolic blood pressure; SFA, saturated fat; TE, total energy.

**Table 2:** Baseline sociodemographic and lifestyle factors and cumulative average dietary intakes in 7752 women according to quintiles of animal protein and plant protein intake ^1^.

	Quintiles of Animal Protein Intake					*p* for Trend	Quintiles of Plant Protein Intake					*p* for Trend
	1	2	3	4	5		1	2	3	4	5	
No. of participants	1550	1550	1551	1550	1551		1550	1550	1551	1550	1551	
Age (year)	38.6 ± 15.3	37.3 ± 13.1	37.7 ± 13.5	37.9 ± 13.7	38.5 ± 14.0	0.662	38.3 ± 14.3	37.8 ± 13.5	37.7 ± 13.4	38 ± 13.6	38.3 ± 15.0	0.746
BMI (kg/m^2^)	21.9 ± 2.9	22.0 ± 3.1	21.9 ± 3	22.1 ± 3.1	22.4 ± 3.5	<0.0001	22.2 ± 3.2	22.0 ± 3.1	21.9 ± 3.2	22.0 ± 3.0	22.2 ± 3.2	0.562
PAL (×BMR)	1.8 ± 0.2	1.7 ± 0.2	1.6 ± 0.2	1.6 ± 0.2	1.5 ± 0.2	<0.0001	1.5 ± 0.2	1.6 ± 0.2	1.7 ± 0.2	1.7 ± 0.2	1.7 ± 0.2	<0.0001
Urban residential area, *n* (%)	220 (14.2%)	365 (23.5%)	560 (36.1%)	747 (48.2%)	1006 (64.9%)	<0.0001	888 (57.3%)	683 (44.1%)	528 (34.0%)	452 (29.2%)	347 (22.4%)	<0.0001
Education level, *n* (%)						<0.0001						<0.0001
Low	1076 (69.4%)	895 (57.7%)	680 (43.8%)	530 (34.2%)	321 (20.7%)		402 (25.9%)	594 (38.3%)	752 (48.5%)	831 (53.6%)	923 (59.5%)	
Medium	463 (29.9%)	625 (40.3%)	794 (51.2%)	914 (59.0%)	990 (63.8%)		950 (61.3%)	844 (54.5%)	733 (47.3%)	677 (43.7%)	582 (37.5%)	
High	11 (0.7%)	30 (1.9%)	77 (5.0%)	106 (6.8%)	240 (15.5%)		198 (12.8%)	112 (7.2%)	66 (4.3%)	42 (2.7%)	46 (3.0%)	
Household income level, *n* (%)						<0.0001						<0.0001
Low	875 (56.5%)	600 (38.7%)	436 (28.1%)	256 (16.5%)	136 (8.8%)		174 (11.2%)	329 (21.2%)	487 (31.4%)	581 (37.5%)	732 (47.2%)	
Medium	426 (27.5%)	504 (32.5%)	486 (31.3%)	456 (29.4%)	296 (19.1%)		297 (19.2%)	471 (30.4%)	486 (31.3%)	467 (30.1%)	447 (28.8%)	
High	182 (11.7%)	281 (18.1%)	374 (24.1%)	455 (29.4%)	476 (30.7%)		484 (31.2%)	406 (26.2%)	344 (22.2%)	303 (19.5%)	231 (14.9%)	
Very high	67 (4.3%)	165 (10.6%)	255 (16.4%)	383 (24.7%)	643 (41.5%)		595 (38.4%)	344 (22.2%)	234 (15.1%)	199 (12.8%)	141 (9.1%)	
Ever and current smoker, *n* (%)	77 (5.0%)	69 (4.5%)	59 (3.8%)	48 (3.1%)	36 (2.3%)	<0.0001	44 (2.8%)	49 (3.2%)	51 (3.3%)	62 (4.0%)	83 (5.4%)	0.0001
Alcohol consumer, *n* (%)	152 (9.8%)	164 (10.6%)	178 (11.5%)	201 (13.0%)	232 (15.0%)	<0.0001	220 (14.2%)	190 (12.3%)	192 (12.4%)	166 (10.7%)	159 (10.3%)	0.0003
SBP (mm Hg)	110.1 ± 12.1	109.0 ± 12.0	110.1 ± 11.9	110.1 ± 11.6	111.0 ± 12.1	0.002	110.9 ± 12.1	109.7 ± 11.7	109.7 ± 11.9	109.2 ± 11.8	110.6 ± 12.2	0.459
TE (kcal/day)	2221.3 ± 576.1	2123.8 ± 515.3	2086.9 ± 478.1	2042.4 ± 480.9	2002.6 ± 532.5	<0.0001	1998.7 ± 549.9	2073.4 ± 484.2	2105.8 ± 468.6	2108.9 ± 516.0	2190.0 ± 571.9	<0.0001
Protein (g/day)	58.2 ± 8.8	59.3 ± 8.0	62.1 ± 8.0	66.6 ± 7.8	78.7 ± 13.4	<0.0001	65.0 ± 15.0	64.1 ± 10.8	63.2 ± 10.7	63.4 ± 10.6	69.2 ± 11.5	<0.0001
Protein (%TE)	11.1 ± 1.6	11.4 ± 1.6	12.0 ± 1.6	12.9 ± 1.6	15.2 ± 2.7	<0.0001	12.6 ± 3.0	12.4 ± 2.2	12.2 ± 2.2	12.2 ± 2.1	13.2 ± 2.3	<0.0001
Animal protein (g/day)	2.2 (0.0, 4.1)	9.5 (7.7, 11.2)	16.5 (14.8, 18.2)	24.1 (21.9, 26.2)	35.8 (31.7, 42.9)	<0.0001	28.9 (21.6, 37.7)	21.9 (16.4, 28.7)	16.4 (10.2, 23.8)	10.3 (5.3, 17.3)	4.9 (1.0, 11.1)	<0.0001
Animal protein (%TE)	0.4 (0.0, 0.8)	1.8 (1.5, 2.2)	3.2 (2.8, 3.6)	4.7 (4.3, 5.2)	7.2 (6.2, 8.7)	<0.0001	5.7 (4.2, 7.6)	4.3 (3.1, 5.7)	3.2 (1.9, 4.7)	2.0 (1, 3.4)	0.9 (0.2, 2.2)	<0.0001
Plant protein (g/day)	54.4 ± 9.0	47.9 ± 8.2	43.6 ± 8.3	40.2 ± 7.7	36.5 ± 8.8	<0.0001	30.9 ± 4.5	38.8 ± 1.5	43.8 ± 1.4	49.3 ± 1.9	59.8 ± 6.5	<0.0001
Plant protein (%TE)	10.4 ± 1.7	9.2 ± 1.6	8.3 ± 1.6	7.7 ± 1.5	7.0 ± 1.7	<0.0001	5.9 ± 0.9	7.4 ± 0.3	8.4 ± 0.3	9.4 ± 0.4	11.5 ± 1.2	<0.0001
CHO (%TE)	71.0 ± 7.7	63.6 ± 7.8	58.9 ± 7.3	54.8 ± 7.3	49.3 ± 8.7	<0.0001	48.3 ± 8.6	56.6 ± 7.2	60.5 ± 8.1	64.2 ± 8.3	68.1 ± 9.1	<0.0001
SFA (%TE)	3.6 ± 2.2	5.6 ± 2.5	6.8 ± 2.2	7.8 ± 2.4	8.9 ± 2.7	<0.0001	9.4 ± 2.9	7.5 ± 2.3	6.4 ± 2.2	5.4 ± 2.2	4.0 ± 2.2	<0.0001
PUFA (%TE)	3.9 (2.4, 6.2)	5.2 (3.4, 8.0)	5.6 (3.8, 8.1)	6.1 (4.1, 8.7)	7.1 (4.9, 9.9)	<0.0001	7.3 (4.9, 10.9)	5.9 (4.0, 8.4)	5.3 (3.5, 8.3)	5.2 (3.2, 7.8)	4.5 (2.9, 6.6)	<0.0001
MUFA (%TE)	6.6 ± 3.5	9.8 ± 3.7	12.0 ± 3.6	13.6 ± 3.7	15.0 ± 4.4	<0.0001	16.3 ± 4.3	13.1 ± 3.5	11.2 ± 3.4	9.3 ± 3.4	7.1 ± 3.5	<0.0001
Dietary fiber (g/day)	12.9 (9.8, 15.8)	10.9 (8.5, 13.8)	9.9 (7.6, 12.4)	9.2 (7.2, 12.2)	9.2 (7.1, 12.6)	<0.0001	8.4 (6.7, 11.2)	8.7 (7.0, 11.3)	9.8 (7.8, 12.2)	11.2 (9.0, 13.6)	14.1 (11.8, 16.9)	<0.0001
Cholesterol (mg/day)	27.6 (4.0, 68.7)	118.2 (71.4, 181.2)	179 (118.4, 260.3)	229.5 (169.6, 322.8)	333.9 (245.3, 460.9)	<0.0001	271.1 (185.6, 387.4)	212.1 (140.0, 307.8)	168.2 (100.3, 270.5)	127.3 (59.9, 218.3)	64.6 (15.0, 160.7)	<0.0001
Sodium (mg/day)	5576 ± 2767	5540 ± 2500	5242 ± 2355	5233 ± 2261	5221 ± 2328	<0.0001	5352 ± 2632	5225 ± 2196	5413 ± 2268	5435 ± 2451	5387 ± 2680	0.220
Calcium (mg/day)	361.7 ± 146.3	363.8 ± 141.3	375.9 ± 157.9	395.9 ± 171.2	471.4 ± 214.7	<0.0001	394.9 ± 201.0	375.2 ± 145.9	383.5 ± 167	396.2 ± 162.0	419.0 ± 181.6	<0.0001
Magnesium (mg/day)	333.4 ± 84.6	302.2 ± 65.0	288.6 ± 60.7	285.2 ± 63.7	292.3 ± 64.0	<0.0001	255.4 ± 52.8	274.5 ± 49.4	294.0 ± 56.1	316.2 ± 63.6	361.5 ± 74.8	<0.0001
Potassium (mg/day)	1544.4 ± 457.3	1495.8 ± 383.0	1507.4 ± 376.8	1584.8 ± 439.6	1784.3 ± 467.3	<0.0001	1567.5 ± 451.7	1509.3 ± 389.6	1551.3 ± 420.6	1580.0 ± 391.8	1708.5 ± 505.8	<0.0001

^1^ Values are presented as the mean ± SD, count (%), or median (IQR). We calculated dietary intake as the energy-adjusted cumulative average of baseline and follow-up data in men and women, respectively. For non-dietary factors, we used the baseline measurement. To test the linear trends, we used linear regression for continuous variables (including the median intake of animal or plant protein as continuous variables in the regression models) and the chi-square with linear-by-linear association tests for categorical variables. BMI, body mass index; BMR, basal metabolic rate; CHO, carbohydrates; IQR, interquartile range; MUFA, monounsaturated fat; PAL, physical activity level; PUFA, polyunsaturated fat; SD, standard deviation; SBP, systolic blood pressure; SFA, saturated fat; TE, total energy.

**Table 3:** Associations between hypertension risk and cumulative average dietary intakes of animal, plant, and total protein in Chinese men and women ^1^.

	Quintiles of Intake in Men					*p* for Trend	Quintiles of Intake in Women					*p* for Trend
	1	2	3	4	5		1	2	3	4	5	
	(*n* = 1401)	(*n* = 1401)	(*n* = 1402)	(*n* = 1401)	(*n* = 1402)		(*n* = 1550)	(*n* = 1550)	(*n* = 1551)	(*n* = 1550)	(*n* = 1551)	
Animal protein												
Median intake (g/day)	1.9	10.7	18.9	27.4	41.0		2.2	9.5	16.5	24.1	35.8	
Cases/person-years	673/13,487	529/15,623	494/14,584	495/14,048	395/10,711		689/14,309	544/17,203	456/15,928	380/15,207	307/11,935	
Model 1	1 (ref)	0.70 (0.62, 0.78)	0.68 (0.60, 0.76)	0.71 (0.63, 0.80)	0.75 (0.66, 0.85)	<0.0001	1 (ref)	0.69 (0.61, 0.77)	0.61 (0.54, 0.69)	0.55 (0.48, 0.62)	0.57 (0.50, 0.66)	<0.0001
Model 2	1 (ref)	0.68 (0.60, 0.76)	0.63 (0.55, 0.71)	0.65 (0.57, 0.74)	0.66 (0.57, 0.76)	<0.00001	1 (ref)	0.70 (0.62, 0.78)	0.61 (0.54, 0.69)	0.53 (0.46, 0.61)	0.56 (0.47, 0.65)	<0.0001
Model 3	1 (ref)	0.75 (0.66, 0.85)	0.73 (0.63, 0.84)	0.77 (0.66, 0.91)	0.78 (0.65, 0.95)	0.137	1 (ref)	0.80 (0.70, 0.90)	0.75 (0.65, 0.87)	0.70 (0.59, 0.83)	0.76 (0.62, 0.93)	0.010
Plant protein												
Median intake (g/day)	37.0	44.8	50.5	57.1	67.7		32.1	38.9	43.7	49.2	58.4	
Cases/person-years	408/10,205	483/15,082	496/15,481	559/14,678	640/13,007		280/11,574	403/16,187	490/16,922	554/16,223	649/13,676	
Model 1	1 (ref)	0.86 (0.76, 0.99)	0.84 (0.73, 0.96)	1.07 (0.93, 1.22)	1.29 (1.13, 1.46)	<0.0001	1 (ref)	1.01 (0.86, 1.18)	1.12 (0.97, 1.31)	1.34 (1.16, 1.56)	1.80 (1.55, 2.08)	<0.0001
Model 2	1 (ref)	0.88 (0.77, 1.01)	0.87 (0.76, 1.00)	1.11 (0.97, 1.28)	1.36 (1.19, 1.56)	<0.0001	1 (ref)	0.98 (0.84, 1.15)	1.08 (0.92, 1.26)	1.33 (1.14, 1.55)	1.71 (1.46, 1.99)	<0.0001
Model 3	1 (ref)	0.83 (0.72, 0.95)	0.78 (0.67, 0.91)	0.93 (0.79, 1.10)	1.11 (0.91, 1.36)	0.031	1 (ref)	0.89 (0.76, 1.05)	0.92 (0.77, 1.08)	1.07 (0.89, 1.28)	1.29 (1.04, 1.59)	0.0003
Total protein												
Median intake (g/day)	60.0	67.6	73.4	79.4	90.5		52.1	58.7	63.4	68.8	79.3	
Cases/person-years	540/12,887	531/15,563	552/16,146	516/13,799	447/10,058		514/14,132	522/17,435	514/17,042	469/15,248	357/10,725	
Model 1	1 (ref)	0.84 (0.74, 0.95)	0.85 (0.75, 0.95)	0.92 (0.82, 1.04)	1.11 (0.97, 1.26)	0.050	1 (ref)	0.82 (0.73, 0.93)	0.81 (0.72, 0.92)	0.85 (0.75, 0.97)	0.95 (0.83, 1.09)	0.590
Model 2	1 (ref)	0.81 (0.72, 0.92)	0.85 (0.75, 0.96)	0.92 (0.81, 1.04)	1.08 (0.94, 1.23)	0.095	1 (ref)	0.85 (0.75, 0.96)	0.85 (0.75, 0.97)	0.88 (0.78, 1.01)	1.02 (0.88, 1.18)	0.773
Model 3	1 (ref)	0.80 (0.71, 0.90)	0.81 (0.72, 0.92)	0.87 (0.76, 0.99)	0.97 (0.84, 1.13)	0.832	1 (ref)	0.85 (0.75, 0.96)	0.83 (0.73, 0.95)	0.85 (0.74, 0.98)	0.97 (0.83, 1.14)	0.692

^1^ Values are presented as HRs (95% CIs), calculated by using Cox proportional hazard analyses. Dietary intakes were calculated as the energy-adjusted cumulative average of baseline and follow-up data. Tests for the linear trend of HRs were conducted by using the median value for each quintile of intake as a continuous variable. Model 1: adjusted for baseline age, BMI, and dietary intake of TE. Model 2: variables adjusted for in Model 1 + residential area, highest education level, household income level, PAL, smoking status, alcohol consumption, and SBP at baseline. Model 3: variables adjusted for in Model 2 + dietary intakes of SFA, PUFA, dietary fiber, sodium, calcium, and magnesium. Mutual adjustment was performed for dietary animal and plant protein. BMI, body mass index; CI, confidence interval; HR, hazard ratio; PAL, physical activity level; PUFA, polyunsaturated fat; SBP, systolic blood pressure; SFA, saturated fat; TE, total energy.

## Data Availability

Publicly available datasets were analyzed in this study. This data can be found here: [https://www.cpc.unc.edu/projects/china] (accessed on 20 January 2022).

## Supplementary Materials

The following is available online at https://www.mdpi.com/article/10.3390/nu14061276/s1, Table S1: Associations between hypertension risk and cumulative average dietary intakes of red meat and white meat protein in Chinese men and women; Table S2: Stratified analyses of protein–hypertension association by BMI, age, smoking and alcohol drinking status in Chinese men and women; Figure S1: Multivariable-adjusted HRs and 95% CIs for the risk of HT according to dietary intakes of red meat and white meat protein on a continuous scale.
