# Supplementary material for: Association between Protein Intake and the Risk of Hypertension among Chinese Men and Women: A Longitudinal Study
Source: Nutrients. 2022 Mar 17;14(6):1276. doi: 10.3390/nu14061276 (PMC8955461; doi:10.3390/nu14061276)
Supplement: Supplementary file 1 [file nutrients-14-01276-s001.zip › nutrients-1606209-supplementary.pdf]

Supplementary materials

Table S1. Associations between hypertension risk and cumulative average dietary intakes of red meat and white meat protein in Chinese men and women[1]

	Quintiles of intake in men					p for trend	Quintiles of intake in women					p for trend
	1	2	3	4	5		1	2	3	4	5	
**Red meat protein**												
Number of participants	1401	1401	1402	1401	1402		1550	1550	1551	1550	1551	
Median intake (g/day)	0	5.0	10.0	15.7	25.6		0	4.0	8.2	13.2	21.2	
Cases/person-years	653/12960	535/15293	494/14635	494/14047	410/11518		669/13432	509/16801	455/15629	404/15945	339/12775	
Model 1	1 (ref)	0.71(0.63, 0.80)	0.68(0.61, 0.77)	0.74(0.66, 0.84)	0.75(0.66, 0.85)	0.0003	1 (ref)	0.64(0.57, 0.72)	0.58(0.51, 0.65)	0.51(0.45, 0.58)	0.59(0.52, 0.67)	<.0001
Model 2	1 (ref)	0.71(0.63, 0.79)	0.64(0.56, 0.72)	0.68(0.60, 0.78)	0.68(0.59, 0.78)	<.0001	1 (ref)	0.67(0.60, 0.75)	0.58(0.51, 0.66)	0.51(0.45, 0.58)	0.58(0.50, 0.68)	<.0001
Model 3	1 (ref)	0.77(0.68, 0.87)	0.76(0.65, 0.87)	0.85(0.72, 0.99)	0.85(0.71, 1.02)	0.863	1 (ref)	0.75(0.67, 0.85)	0.70(0.61, 0.82)	0.66(0.56, 0.78)	0.78(0.64, 0.93)	0.044
**White meat protein**												
Number of participants[2]	2588	1104	1105	1105	1105		2769	1245	1246	1246	1246	
Median intake (g/day)	0	2.2	5.3	9.1	16.9		0	1.9	4.6	7.9	14.7	
Cases/person-years	1048/21270	413/14336	399/12390	380/11550	346/8907		978/21695	433/16049	358/13865	320/13209	287/9764	
Model 1	1 (ref)	0.56(0.50, 0.63)	0.64(0.57, 0.72)	0.66(0.59, 0.74)	0.80(0.71, 0.90)	0.0012	1 (ref)	0.57(0.51, 0.64)	0.59(0.52, 0.67)	0.58(0.51, 0.65)	0.69(0.61, 0.79)	<.0001
Model 2	1 (ref)	0.55(0.49, 0.62)	0.63(0.56, 0.71)	0.64(0.57, 0.72)	0.74(0.65, 0.85)	0.0002	1 (ref)	0.59(0.52, 0.66)	0.60(0.53, 0.68)	0.59(0.51, 0.67)	0.73(0.63, 0.84)	<.0001
Model 3	1 (ref)	0.58(0.52, 0.66)	0.66(0.59, 0.75)	0.67(0.59, 0.77)	0.76(0.66, 0.88)	0.018	1 (ref)	0.64(0.57, 0.73)	0.67(0.59, 0.77)	0.66(0.57, 0.76)	0.83(0.71, 0.97)	0.059

[1] Values are presented as HRs (95% CIs), calculated by using Cox proportional hazard analyses. Dietary intakes were calculated as the energy-adjusted cumulative average of baseline and follow-up data. Tests for the linear trend of HRs were conducted by using the median value for each quintile of intake as a continuous variable.

[2] For white meat protein, because of the relatively low intake, people with a zero intake were set as the reference group, and the others were divided into four groups by quartiles.

Model 1: adjusted for baseline age, BMI and dietary intake of TE. Model 2: variables adjusted for in Model 1 + residential area, highest education level, household income level, PAL, smoking status, alcohol consumption, and SBP at baseline. Model 3: variables adjusted for in Model 2 + dietary intakes of plant protein, SFA, PUFA, dietary fiber, sodium, calcium and magnesium. Mutual adjustment was performed for red meat and white meat protein.

BMI, body mass index; CI, confidence interval; HR, hazard ratio; PAL, physical activity level; PUFA, polyunsaturated fat; SBP, systolic blood pressure; SFA, saturated fat; TE, total energy.

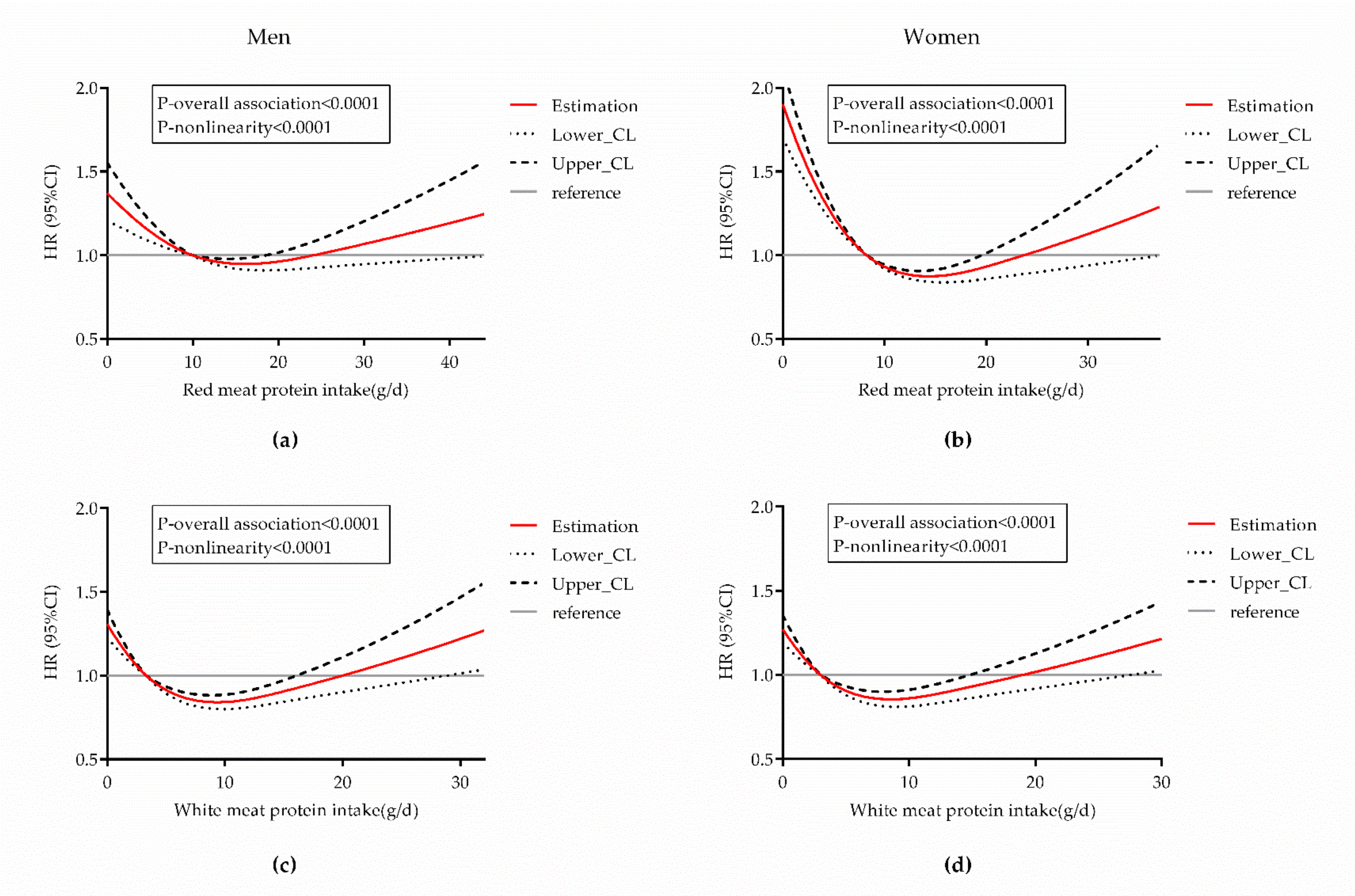

Figure S1. Multivariable-adjusted HRs and 95% CIs for the risk of HT according to dietary intakes of red meat and white meat protein on a continuous scale

CI, confidence interval; CL, confidence limit; HT, hypertension.

(**a**) Association between red meat protein intake and HT risk in men; (**b**) Association between red meat protein intake and HT risk in women; (**c**) Association between white meat protein intake and HT risk in men; (**d**) Association between white meat protein intake and HT risk in women. Red solid lines represent the multivariable-adjusted HRs, with black dotted lines representing the 95% CIs. Gray horizontal solid lines represent the references that were set at the median intakes, corresponding to an HR of 1.0. Analyses were adjusted for a series of variables according to Model 3.

Table S2. Stratified analyses of protein–hypertension association by BMI, age, smoking and alcohol drinking status in Chinese men and women[1]

	Quintiles of intake in males					P for trend		Quintiles of intake in females					P for trend
	1	2	3	4	5			1	2	3	4	5	
**Animal protein**													
BMI<24kg/m² (*n* = 5609)							BMI<24kg/m² (*n* = 5937)						
HR (95%CI)	1(ref)	0.76(0.66,0.87)	0.74(0.63,0.87)	0.81(0.67,0.98)	0.81(0.65,1.01)	0.255	HR (95%CI)	1(ref)	0.78(0.67,0.90)	0.74(0.62,0.89)	0.71(0.58,0.87)	0.70(0.55,0.91)	0.0069
BMI≥24kg/m² (*n* = 1398)							BMI≥24kg/m² (*n* = 1815)						
HR (95%CI)	1(ref)	0.72(0.53,0.98)	0.73(0.52,1.01)	0.67(0.47,0.95)	0.69(0.47,1.02)	0.2271	HR (95%CI)	1(ref)	0.88(0.69,1.14)	0.86(0.65,1.14)	0.75(0.54,1.03)	0.94(0.66,1.33)	0.8233
P-interaction						0.162	*P*-interaction						0.003
Age<50y (*n* = 5573)							Age<50y (*n* = 6124)						
HR (95%CI)	1(ref)	0.78(0.68,0.91)	0.72(0.61,0.86)	0.78(0.64,0.96)	0.81(0.64,1.02)	0.3125	HR (95%CI)	1(ref)	0.85(0.73,0.99)	0.79(0.66,0.95)	0.76(0.61,0.94)	0.78(0.6,1.02)	0.0642
Age≥50y (*n* = 1434)							Age≥50y (*n* = 1628)						
HR (95%CI)	1(ref)	0.68(0.54,0.87)	0.79(0.61,1.02)	0.82(0.61,1.10)	0.78(0.55,1.10)	0.4954	HR (95%CI)	1(ref)	0.70(0.56,0.88)	0.7(0.54,0.9)	0.61(0.46,0.81)	0.75(0.54,1.04)	0.1298
P-interaction						0.443	*P*-interaction						0.749
Non-smoker (*n* = 2619)							Non-smoker (*n* = 7463)						
HR (95%CI)	1(ref)	0.88(0.70,1.11)	0.74(0.57,0.96)	0.81(0.60,1.07)	0.85(0.61,1.18)	0.4706	HR (95%CI)	1(ref)	0.81(0.71,0.92)	0.75(0.65,0.88)	0.69(0.58,0.83)	0.75(0.61,0.92)	0.0073
Former or current smoker (*n*=4388)							Ever or current smoke (*n* = 289)						
HR (95%CI)	1(ref)	0.71(0.61,0.82)	0.73(0.61,0.87)	0.77(0.63,0.94)	0.76(0.60,0.96)	0.2136	HR (95%CI)	1(ref)	0.60(0.35,1.03)	0.67(0.37,1.22)	0.67(0.32,1.42)	1.03(0.42,2.51)	0.9229
P-interaction						0.746	*P*-interaction						0.511
Non-drinker (*n* = 2686)							Non-drinker (*n* = 6825)						
HR (95%CI)	1(ref)	0.76(0.61,0.94)	0.78(0.60,1.00)	0.78(0.58,1.03)	0.80(0.57,1.11)	0.3751	HR (95%CI)	1(ref)	0.80(0.70,0.92)	0.73(0.62,0.85)	0.67(0.56,0.81)	0.74(0.6,0.92)	0.0064
Drinker (*n* = 4321)							Drinker (*n* = 927)						
HR (95%CI)	1(ref)	0.74(0.64,0.87)	0.71(0.60,0.85)	0.77(0.63,0.94)	0.76(0.60,0.96)	0.1672	HR (95%CI)	1(ref)	0.70(0.48,1.01)	0.82(0.54,1.26)	0.77(0.47,1.25)	0.79(0.44,1.41)	0.61
P-interaction						0.796	*P*-interaction						0.857
**Plant protein**													
BMI<24kg/m² (*n* = 5609)							BMI<24kg/m² (*n* = 5937)						
HR (95%CI)	1(ref)	0.81(0.69,0.96)	0.77(0.64,0.92)	0.94(0.77,1.15)	1.19(0.94,1.51)	0.0088	HR (95%CI)	1(ref)	0.94(0.77,1.16)	0.98(0.79,1.22)	1.21(0.95,1.54)	1.46(1.11,1.92)	<.0001
BMI≥24kg/m² (*n* = 1398)							BMI≥24kg/m² (*n* = 1815)						
HR (95%CI)	1(ref)	0.83(0.63,1.10)	0.76(0.56,1.02)	0.85(0.60,1.19)	0.85(0.56,1.28)	0.6172	HR (95%CI)	1(ref)	0.87(0.67,1.13)	0.9(0.69,1.18)	0.92(0.68,1.24)	1.2(0.85,1.69)	0.1773

P-interaction						0.379	*P*-interaction						0.608
Age<50y (*n* = 5573)							Age<50y (*n* = 6124)						
HR (95%CI)	1(ref)	0.85(0.71,1.00)	0.83(0.69,1.00)	0.98(0.80,1.20)	1.09(0.85,1.40)	0.1009	HR (95%CI)	1(ref)	0.97(0.79,1.19)	1.04(0.83,1.29)	1.22(0.96,1.56)	1.5(1.13,1.98)	0.0001
Age≥50y (*n* = 1434)							Age≥50y (*n* = 1628)						
HR (95%CI)	1(ref)	0.85(0.66,1.09)	0.66(0.50,0.87)	0.85(0.63,1.14)	1.23(0.86,1.76)	0.148	HR (95%CI)	1(ref)	0.79(0.61,1.02)	0.8(0.61,1.05)	0.95(0.71,1.27)	1.13(0.81,1.58)	0.0964
P-interaction						0.560	*P*-interaction						0.128
Non-smoker (*n* = 2619)							Non-smoker (*n* = 7463)						
HR (95%CI)	1(ref)	0.79(0.61,1.01)	0.81(0.62,1.06)	1.03(0.77,1.39)	1.15(0.80,1.64)	0.0884	HR (95%CI)	1(ref)	0.84(0.71,0.99)	0.87(0.74,1.04)	1.03(0.85,1.24)	1.19(0.96,1.49)	0.003
Former or current smoker (*n* = 4388)							Ever or current smoke (*n* = 289)						
HR (95%CI)	1(ref)	0.84(0.71,1.00)	0.76(0.63,0.92)	0.89(0.72,1.09)	1.10(0.86,1.41)	0.1262	HR (95%CI)	1(ref)	2.95(1.10,7.90)	3.33(1.15,9.61)	2.81(0.97,8.17)	6.12(1.94,19.38)	0.0034
P-interaction						0.832	*P*-interaction						0.687
Non-drinker (*n* = 2686)							Non-drinker (*n* = 6825)						
HR (95%CI)	1(ref)	0.67(0.52,0.86)	0.52(0.39,0.68)	0.68(0.50,0.93)	0.78(0.54,1.11)	0.8998	HR (95%CI)	1(ref)	0.88(0.74,1.04)	0.96(0.8,1.15)	1.09(0.89,1.33)	1.29(1.03,1.62)	0.0006
Drinker (*n* = 4321)							Drinker (*n* = 927)						
HR (95%CI)	1(ref)	0.88(0.74,1.05)	0.92(0.77,1.11)	1.03(0.84,1.27)	1.26(0.98,1.61)	0.0129	HR (95%CI)	1(ref)	0.95(0.62,1.45)	0.64(0.4,1.03)	0.96(0.57,1.62)	1.36(0.74,2.49)	0.1303
P-interaction						0.392	*P*-interaction						
													0.601
**Total protein**													
BMI<24kg/m² (*n* = 5609)							BMI<24kg/m² (*n* = 5937)						
HR (95%CI)	1(ref)	0.84(0.74,0.97)	0.83(0.72,0.96)	0.94(0.81,1.09)	1.02(0.86,1.20)	0.5939	HR (95%CI)	1(ref)	0.84(0.73,0.97)	0.83(0.71,0.96)	0.82(0.69,0.96)	1(0.82,1.21)	0.5976
BMI≥24kg/m² (*n* = 1398)							BMI≥24kg/m² (*n* = 1815)						
HR (95%CI)	1(ref)	0.60(0.45,0.80)	0.68(0.52,0.90)	0.60(0.45,0.80)	0.76(0.57,1.03)	0.4336	HR (95%CI)	1(ref)	0.85(0.66,1.11)	0.86(0.66,1.12)	0.95(0.73,1.23)	1.01(0.76,1.35)	0.5246
P-interaction						0.190	*P*-interaction						0.091
Age<50y (*n* = 5573)							Age<50y (*n* = 6124)						
HR (95%CI)	1(ref)	0.81(0.70,0.94)	0.80(0.69,0.93)	0.87(0.74,1.01)	0.96(0.81,1.15)	0.9749	HR (95%CI)	1(ref)	0.86(0.73,1.00)	0.86(0.73,1.01)	0.89(0.76,1.06)	1(0.81,1.23)	0.967
Age≥50y (*n* = 1434)							Age≥50y (*n* = 1628)						
HR (95%CI)	1(ref)	0.80(0.64,1.01)	0.89(0.70,1.13)	0.88(0.69,1.12)	1.05(0.80,1.37)	0.5823	HR (95%CI)	1(ref)	0.84(0.68,1.03)	0.77(0.62,0.97)	0.75(0.59,0.96)	0.96(0.74,1.24)	0.632
P-interaction						0.799	*P*-interaction						0.245
Non-smoker (*n* = 2619)							Non-smoker (*n* = 7463)						
HR (95%CI)	1(ref)	0.87(0.70,1.09)	0.83(0.66,1.05)	0.98(0.78,1.23)	1.04(0.81,1.35)	0.4728	HR (95%CI)	1(ref)	0.84(0.74,0.95)	0.81(0.71,0.93)	0.84(0.73,0.96)	0.94(0.8,1.11)	0.449
Former or current smoker (*n* = 4388)							Ever or current smoke (*n* = 289)						
HR (95%CI)	1(ref)	0.77(0.67,0.89)	0.81(0.70,0.94)	0.82(0.70,0.97)	0.95(0.79,1.13)	0.846	HR (95%CI)	1(ref)	1.00(0.57,1.78)	1.09(0.58,2.03)	0.88(0.44,1.73)	1.51(0.77,2.98)	0.2964

P-interaction						0.587	*P*-interaction						0.462
Non-drinker (*n* = 2686)							Non-drinker (*n* = 6825)						
HR (95%CI)	1(ref)	0.85(0.69,1.05)	0.85(0.68,1.06)	0.90(0.72,1.13)	0.93(0.72,1.21)	0.7445	HR (95%CI)	1(ref)	0.79(0.69,0.90)	0.78(0.68,0.9)	0.8(0.7,0.93)	0.93(0.78,1.1)	0.3991
Drinker (*n* = 4321)							Drinker (*n* = 927)						
HR (95%CI)	1(ref)	0.77(0.66,0.90)	0.80(0.68,0.93)	0.85(0.72,0.99)	0.97(0.82,1.16)	0.7788	HR (95%CI)	1(ref)	1.25(0.87,1.78)	1.21(0.82,1.8)	1.26(0.82,1.94)	1.27(0.79,2.06)	0.4132
P-interaction						0.682	*P*-interaction						0.156

[1]All the analyses were multi-variable adjusted based on Model 3s as in Table 3.

BMI, body mass index; CI, confidence interval; HR, hazard ratio.
